# Low birth weight among term newborns in Wolaita Sodo town, South Ethiopia: a facility based cross-sectional study

**DOI:** 10.1186/s12884-018-1789-y

**Published:** 2018-05-11

**Authors:** Samson Kastro, Tsegaye Demissie, Bereket Yohannes

**Affiliations:** 1 School of Public Health, Wolaita Sodo University, Wolaita Sodo, Ethiopia; 2 School of Public Health, Hawassa University, Hawassa, Ethiopia; 3 Centre for International Health, University of Bergen, Bergen, Norway

**Keywords:** Low birth weight, Term singletons, Wolaita Sodo, Ethiopia

## Abstract

**Background:**

In low income countries, many low birth weight newborns often miss the chance for survival sooner or later. Others who survive would also face increased risks in later life. Though not adequately documented in Ethiopia, maternal factors pose the main risk. This study was aimed to estimate the proportion of low birth weight among term singletons without congenital malformations and factors associated with it in Wolaita Sodo town in South Ethiopia.

**Methods:**

We did a facility based survey involving 432 postpartum women with their term newborns. Data was collected through face to face interview from March to April in 2016. The outcome measure was newborn birth weight. Bivariate logistic regression was applied to look for crude associations. Multivariate logistic regression analysis was done to adjust for potential confounders to identify independent predictors. Adjusted Odds Ratio (AOR) and 95% confidence intervals (CI), and statistical significance at *P* < 0.05 were reported.

**Results:**

The proportion of term low birth weight was 8.1% in the study area. Women who had less education (AOR = 6.23; 95% CI = 1.68, 23.1), house wives (AOR = 5.85; 95% CI = 1.40, 24.3) and not frequently consuming fruits during pregnancy (AOR 11.3; 95% CI = 1.98, 64.9) had a higher risk of having term low birth weight newborns. We documented a lesser odds of those from rural settings to have low birth weight newborns as compared to their counter urban equivalents (AOR = 0.06; 95% CI = 0.006, 0.6).

**Conclusions:**

Dietary counselling to pregnant mothers specific diet and nutrition including fruit diets in particular might contribute to reduce the risk of term low birth weight. Better education might have enabled women to prefer diets and their job engagements might also have capacitated them to decide on dietary preferences.

**Electronic supplementary material:**

The online version of this article (10.1186/s12884-018-1789-y) contains supplementary material, which is available to authorized users.

## Background

If a newborn weighs less than 2500 g at birth, it is termed as low birth weight (LBW) [1]. A low birth weight carries an increased risk of death on the newborns early in life or exposes to multiple health and development challenges later. The burden of immediate health problems on low birth weight newborns has been relatively widely documented in many low income countries with national demographic surveys [2].

An estimated 13% all babies each year in Sub-Saharan Africa (SSA) are born LBW [3–6]. According to the Ethiopian Demographic and Health Surveys an estimated 13–11% of all babies in the country are born low birth weight [7, 8]. The proportions have been documented with a wide variation across the different settings in the country. The prevalence has so far documented as low as 6.3 to 28.3% across Ethiopia [7–13]. Thus the national prevalence of low birth weight has remained high in Ethiopia.

In Ethiopia, most commonly used data on birth weight of the newborns are often based on national survey indirect indicators. However, the national data is known for its limitations including sampling strategies which would not be assumed to be representative of the socio-economic disparities and cultural diversities across the country. Though the proportion of skilled childbirth attendance is increasing in Ethiopia, we lack adequate evidence on maternal factors which would potentially predict birth outcomes of the newborns. This is so limited particularly in South Ethiopia which is characterized by a diverse culture and dietary practices. Thus the proportion of low birth weight in health facilities has been least documented in South Ethiopia.

A densely populated Wolaita area in south Ethiopia [14–19], many households often suffer from chronic food insecurity [20]. Thus even at times of crop harvest, many children in the area remain undernourished. However, we have very limited evidence on child health and nutritional status predictors particularly attached to maternal health during pregnancy and child births. Thus the main aim of this study was to estimate the proportion of low birth weight among term singletons that were born without congenital malformations in Wolaita Sodo town in South Ethiopia. It was also further aimed to identify socio-demographic and maternal factors associated with the outcome. The findings might help for local community based interventions to improve birth outcomes, and also expected to add on the existing knowledge.

## Methods

### Study setting

Wolaita Sodo town is an administrative capital for the Wolaita zonal administration in South Ethiopia. It is located at 380 km south from Addis Ababa. The town has 3 sub-cities; 11 lower administrative units. It had a census projected population of 110,659; 48% were female.

One government hospital and another private one, 3 health centres, 11 health posts and 21 other private health facilities deliver health services to the population in the town. Out of all facilities; only health centres and hospitals are staffed to attend skilled childbirth [21]. Based on the local health service administrators’ report for 2014/15, about 5000 childbirths were attended in government’s health facilities at Wolaita Sodo town.

### Study design and period

We did a facility based cross-sectional study involving postpartum mothers and their newborns through birth records from March to April in 2016.

### Population and sampling

All postpartum mothers and their newborns in government health facilities in Wolaita Sodo town were the source population. Consecutively recruited 432 mother-newborn-pairs were studied. Term newborns (born at 37 weeks or later of gestation) and singletons were included. Birth outcomes such as congenital malformations, multiple births, and stillbirths, and postpartum mothers with unknown last normal menstrual period were excluded.

A sample size of 451 was calculated with the following assumptions: 22.5% expected proportion of low birth outcome which was taken from similar Ethiopian study [13], 95% confidence level, 5% margin of error, and 10% none-response consideration. It was proportionally allocated to government health facilities in Wolaita Sodo town based on number of previous childbirth attendance. Finally all eligible postpartum mother- to-child pairs were consecutively recruited until subsamples for the facilities and the total sample for the study were achieved.

### Variables and measurements

#### Outcome

Birth weight of a term singleton newborn was an outcome measure. It was considered low birth weight if the newborn weighed less than 2500 g. Birth weight measurement was taken within an hour after birth [1].

### Exposure variables and covariates

Socio-demographic and socio-economic: maternal age, educational status, occupation, residence, family income and newborn’s sex [22].

Diets and supplements: consumption of any additional meals and supplements such as iron and folic acid during pregnancy [23].

Maternal morbidity and previous obstetric histories: history of hypertension, diabetes mellitus and infections during pregnancy [24], pregnancy intervals, number of childbirth, low birth weight, abortions, and stillbirths [24].

Maternal health service utilization: antenatal care (ANC), gestational age at first ANC visit, and dietary counselling in ANC [22, 23]. Whereas, a woman needs to make at least four ANC visits during pregnancy [25, 26].

Maternal nutritional status: Mid Upper Arm Circumference (MUAC) of mothers. Whereas, MUAC less than 23 cm centimetres defined the women as undernourished [27].

### Data collection

We adopted a structured questionnaire from relevant articles and related literatures (Additional file 1) [7, 13]. The questionnaire was pretested in 23 respondents which were later not included to the main study. Four data collectors were trained on: the different modules of the questionnaire, participants’ selection, maternal MUAC and birth weight measurements, and ethics.

Anthropometric measures were standardised for Technical Errors against an expert measurer. Weights of newborns were measured within an hour after birth with a digital weight scale. The MUAC of mothers was measured by using a none-stretchable tape.

### Statistical analysis

Data were entered, cleaned and analyzed by using SPSS version 20. Descriptive statistics were done for the main variables. Through binary logistic regression analysis we selected exposure variables with a crude association to the outcome. The multivariate candidate variables were prioritized by using a yardstick cut-off point for statistical significance on bivariate analysis. Thus exposures and covariates with *p*-value less than 0.25 were taken for multivariate analysis. Finally, a multivariate logistic regression analysis was done to control for potential confounders and identify independent predictors of the outcome. Accordingly, we reported AORs as effect measures with 95% CIs, and statistical significance declared at *p*-value < 0.05.

## Results

### Socio-demographic characteristics

Overall, 432 mother-newborn-pairs were involved in the study with a response rate of 95.8%. About nine in ten, 387 (89.6%) were aged 20–34 years. The majority 374 (86.6%) had some level of formal education and 187 (43.3%) were housewives. About two-third, 291(67.4%) were urban and a third were rural residents (Table 1).

**Table 1:** Socio-demographic characteristics of mothers and newborns involved in low birth weight study in health facilities at Wolaita Sodo town in South Ethiopia, March 2016

Variables (*n* = 432)		Frequency	Percent
Age	< 20	24	5.6
	20–34	387	89.6
	35+	21	4.9
Marital status	Married	408	94.4
	Unmarried	24	5.6
Educational status	No formal education	58	13.4
	Primary School (1–8)	143	33.1
	Secondary School (9–12)	119	27.5
	College and above	112	25.9
Occupation	Housewife	187	43.3
	Government employee	89	20.6
	Private employee	32	7.4
	Merchant	92	21.3
	Others^a^	32	7.4
Residence	Rural	141	32.6
	Urban	291	67.4
Average monthly income	≤810 ($ ≤ 37.5)	33	7.6
	810–1296 ($37.5–60)	112	25.9
	1297–2591 ($61–120)	121	28
	≥2592 ($ ≥ 120)	166	38.4
Sex of the newborn	Male	222	51.4
	Female	210	48.6

^a^Students, daily labourers

### Maternal obstetric and health service utilization

One hundred eighty (41.7%) of mothers were primiparous. About a third, 83 (32.9%) of the women gave birth to the current newborn within two years (≤24 months) after a previous child birth. Thirty four mothers (7.9%) had previous abortion and 18 (4.2%) had still birth. One hundred twenty-five (28.9%) mothers had some medical problems during recent pregnancy. The majority, 396 (91.7%) had at least a visit for antenatal during recent pregnancy; 376 (94.9%) of made their first visit during first trimester of gestation. A little higher than half, 210 (53%) had at least four ANC visits and 290 (73.2%) mothers affirmed for getting dietary advice during ANC follow-up (Table 2).

**Table 2:** Obstetric history, morbidity and health service utilization of mothers who visited health facilities for child birth at Wolaita Sodo town in South Ethiopia, March 2016

Variables (*n* = 432)		Frequency	Percentage
Parity	1	180	41.7
	2–4	223	51.6
	5+	29	6.7
Birth interval in months	≤24	83	32.9
	> 24	169	67.1
History of abortion	Yes	34	7.9
	No	398	92.1
History of stillbirth	Yes	18	4.2
	No	414	95.8
History of having small baby	Yes	0	0
	No	391	90.5
	Not specified	41	9.5
Medical Illnesses during pregnancy	Yes	125	28.9
	No	307	71.1
History of hypertension	Yes	8	1.9
	No	424	98.1
History of diabetes mellitus	Yes	4	0.9
	No	428	99.1
ANC visits for recent pregnancy	Yes	396	91.7
	No	36	8.3
ANC visits	1–3	186	47.0
	4+	210	53.0
Trimester for the first ANC visit	1st	376	94.9
	3rd	20	5.1
Dietary counselling during ANC visit	Yes	290	73.2
	No	106	26.8

### Maternal nutritional status

Based on current findings, 166 (38.4%) mothers had MUAC less than 23 cm (undernourished). Three hundred fifty-five (92%) took iron and folic acid supplements at least once and 264 (61.1%) got less than 90 tablets during recent pregnancy. About two-third, 293 (67.8%) consumed at least one additional meal to their usual meals. Mothers involved in this study frequently consumed cereals 258 (59.7%) from food groups (Table 3).

**Table 3:** Dietary intake and supplements pregnancy, and nutritional status of mothers involved in the study at Wolaita Sodo town in South Ethiopia, March 2016

Variables (*n* = 432)		Frequency	Percent
Cereals	Yes	258	59.7
	No	174	40.3
Starchy roots and tubers	Yes	47	10.9
	No	385	89.1
Legumes	Yes	91	21.1
	No	341	78.9
Dairy products	Yes	34	7.9
	No	398	92.1
Meat	No	432	100
Fish and poultry	No	432	100
Vegetables	Yes	38	8.8
	No	394	91.2
Fruits and nuts	Yes	43	10.0
	No	389	90.0
Egg	Yes	2	0.5
	No	430	99.5
Additional meal during pregnancy	Yes	293	67.8
	No	139	32.2
Took iron supplements	Yes	355	92.0
	No	31	8.0
Number of iron tablets taken	< 90	264	61.1
	90–180	91	21.1
	Unknown	77	17.8
MUAC of the mother	< 23 cm	166	38.4
	23+ cm	266	61.6

### Proportion and predictors of term low birth weight

The proportion of term low birth weight in this study was 8.1% (35/432). The mean weight of the newborns was 3532 g with standard deviation of 565. Mothers with less formal educational status and housewives had about six times at higher risk of giving birth to low birth weight newborns (AOR = 5.86; 95% CI: 1.64, 20.9) and (AOR = 5.41; 95% CI: 1.37,21.3). Mothers who consumed fruits rarely (less frequent than daily) had also a higher risk to give birth to term LBW (AOR 13.9; 95% CI: 2.29, 84.6). On the other hand, rural residents had a lower risk of giving birth to low birth weight newborns as compared to those from the nearby urban dwellers (AOR = 0.06; (95% CI = 0.006, 0.6) (Table 4).

**Table 4:** Factors associated with low birth weight among term newborns in health facilities at Wolaita Sodo town in South Ethiopia, March 2016

Variables (*n* = 432)	LBW		COR (95% CI)	AOR (95% CI)
	Yes (%)	No (%)		
Age				
< 25	11 (11.2)	87 (88.8)	1.63 (0.77,3.46)	1.53 (0.31,7.49)
25+	24 (7.2)	310 (92.8)	1	1
Marital status				
Married	30 (7.4)	378 (92.6)	0.30 (0.10,0.86)	0.08 (0.006,1.18)
Other	5 (20.8)	19 (79.2)	1	1
Educational status				
Primary and below	23 (11.4)	178 (88.6)	2.36 (1.14,4.87)	5.86(1.64,20.9)*
Secondary and above	12 (5.2)	219 (94.8)	1	1
Occupation				
Housewife	20 (10.8)	166 (89.2)	2.08 (1.03,4.22)	5.41(1.37,21.3)*
Others	15 (6.1)	231 (93.9)	1	1
Residence				
Rural	16 (11.3)	125 (88.7)	1.85 (0.92,3.73)	0.06 (0.006,0.6)*
Urban	19 (6.5)	272 (93.5)	1	1
Average monthly income				
≤ 810	6 (18.2)	27 (81.8)	3.88 (1.28,11.8)	2.55(0.19,34.09)
810–1296	12 (10.7)	100 (89.3)	2.09 (0.85–5.15)	0.88(0.09,8.02)
1297–2591	8 (6.6)	113 (93.4)	1.23 (0.46–3.30)	2.44(0.61,9.69)
≥ 2592	9 (5.4)	157 (94.6)	1	1
Parity				
1	22 (12.2)	158 (87.8)	2.56 (1.25,5.23)	1.95 (0.47,7.99)
2+	13 (5.2)	239 (94.8)	1	1
Medical conditions				
Yes	6 (4.8)	119 (95.2)	1	1
No	29 (9.4)	278 (90.6)	2.07 (0.84,5.11)	0.67 (0.18,2.52)
Dietary counselling				
Yes	17 (5.9)	273 (94.1)	1	1
No	12 (11.3)	94 (88.7)	2.05 (0.94,4.45)	3.08 (0.64,14.77)
Number of additional meals				
1	2 (2.8)	70 (97.2)	1	1
2+	21 (9.5)	200 (90.5)	3.67 (0.84,16.07)	3.5 (0.55,22.59)
Iron tablets consumed				
< 90	26 (9.8)	238 (90.2)	3.2 (0.94,10.85)	0.56 (0.13,2.46)
≥ 90	3 (3.3)	88 (96.7)	1	1
MUAC				
< 23 cm	15 (9)	151 (91)	1.22 (0.61,2.46)	3.15 (0.95,10.44)
≥ 23 cm	20 (7.5)	246 (92.5)	1	1
Cereals				
Daily	27 (10.5)	231(89.5)	1	1
< Daily	8 (4.6)	166 (95.4)	2.42 (1.07,5.47)	2.89 (0.66,12.74)
Fruits				
Daily	9 (20.9)	34 (79.1)	1	1
< Daily	26 (6.7)	363 (93.3)	3.69 (1.60,8.52)	13.9(2.29,84.6)*

*statistically significant at *p* < 0.05

## Discussion

Evidence shows that birth weight is a good summary measure of multifaceted public health problems. Thus often used to indicate long-term maternal malnutrition, ill health, and poor health care during pregnancy [8]. This study documented that about 8.1% of term childbirths in government health facilities in Wolaita Sodo town ended up with low birth weight during the study period.

We observed a slightly lesser proportion of LBW in current study as compared to similar studies in Northern Ethiopia (10%) and elsewhere (10.6%) [28, 29]. It was also lower than similar study findings from Pakistan (9.9%) and Kenya (12.3%) [5, 30]. The main possible reason for the slight variation might be due to our exclusion of preterm newborns; whereas, the above studies included preterm babies. Conversely, we found a higher proportion of Term LBW compared to other studies in Ethiopia (6.3%), Iran (6.8%), and Egypt (7.3%) [10, 11, 31, 32].

In consistent with studies from India, Tanzania, and Sudan, lower level of maternal education was documented in this study as a positive predictor of low birth weight [4, 6, 33]. A better formal education might have improved mothers’ perceptions and dispositions in proper dietary habits during pregnancy and health service utilization. On the other hand, it might be assumed that the less educated would tend to be less informed on key dietary recommendations and health care during pregnancy [34].

Unlike many other study findings, this study documented that rural residents had a lower risk to have low birth weight babies as compared to those from nearby urban settings. This finding disagrees with similar study findings from Tigray (Northern Ethiopia) and Bale (South east Ethiopia) hospitals in Ethiopia [10, 11, 35] and also findings from Ghana [36].These might possibly be due to less representation of our sample for rural and urban groups which made our finding with a higher uncertainty (a very wide confidence interval for AORs). It might also be due to socioeconomic and other differences. The current finding agrees with a study in Jimma west Ethiopia which could be homogenous in many aspects to our settings [13].

Housewives had much higher odds of giving birth to low birth weight babies as compared to those who had some occupational engagements. This finding is in difference with similar studies South East in Ethiopia as well as Tanzania [6, 34, 35]. On the other hand, in favour of our finding, a study from Iran revealed an increased risk of low birth weight among housewives as compared to employees [37]. This might imply that housewives are mostly less educated, and often disadvantaged in accessing relevant health messages [7, 8, 11]. This might have affected their perceptions in dietary preferences and health service utilization. Furthermore, our study setting, Wolaita area is known for asset poverty; many households in the area are often food insecure [17, 20].

Mothers who did not consume fruits daily during pregnancy had much higher odds of giving birth to low birth weight as compared to those who did so. This was in agreement with a similar study in Nepal [38]. This might be due to a complementary nutritional benefits of fruits as main food sources for micronutrients; thus if particularly consumed in the first trimester, they could enhance organ development for the foetus. Some studies elsewhere in Ethiopia, Bangladesh, Indonesia and Nepal have documented a significant association between low MUAC measures of mothers and term low birth weight in their newborns [11, 39]; however our data did not imply such association. We recommend further studies to evaluate the effect of maternal undernutrition on newborn birth weight.

## Study limitations

This study was limited to health facilities in scope and used a crossectional data. Thus mainly maternal side predictors of term low birth weight were assessed. Moreover, the time allotted for recruiting participants was limited though we assumed standard procedures of sample size calculation. The estimate might be better representative if a longitudinal follow-up data were used. Certain level of recall bias was expected with regard to menstrual dates and dietary habits; health workers conscious on cultural issues collected the data to reduce recall bias. Though all mothers attending delivery service with ANC follow up were ahead screened for HIV, we could not access this information due to limitations out of our scope. We have no data on other covariates such as food security and a reliable wealth data.

## Conclusions

Though it would or would not be possible to bring all child bearing or potential mothers with less education to school; alternative opportunities of empowering women could be done. Occupational engagements of the women might also contribute to empower them on decision making capacities particularly on diet and health care.

Skilled counselling to diet during pregnancy including intake of fruits would benefit the women. Formal education when feasible and alternatively equivalent approaches targeting on diet and health care utilization would benefit. Moreover, we recommend community based data of birth weight through longitudinal studies for a better estimation of the prevalence of low birth weight.

## Additional file

Additional file 1: Questionnaire: Term low birth weight study, Wolaita Sodo. (DOCX 23 kb)

## Abbreviations

ANC: Antenatal care
AOR: Adjusted Odds Ratio
BMI: Body mass index
CI: Confidence interval
CIH: Centre for International Health
COR: Crude odds ratio
EDHS: Ethiopian Demographic and Health Survey
ETB: Ethiopian Birr
IFA: Iron and folic acid
LBW: Low birth weight
MUAC: Mid-Upper Arm Circumference
NORHED: Norwegian Programme for Capacity Development in Higher Education and Research for Development
SENUPH: South Ethiopia Network of Universities in Public Health
SPSS: Statistical Package for Social Sciences
SSA: Sub-Saharan Africa
UoB: University of Bergen
WSU: Wolaita Sodo University
