# Supplementary material for: Low birth weight among term newborns in Wolaita Sodo town, South Ethiopia: a facility based cross-sectional study
Source: BMC Pregnancy Childbirth. 2018 May 11;18:160. doi: 10.1186/s12884-018-1789-y (PMC5948808; doi:10.1186/s12884-018-1789-y)
Supplement: Supplementary file 1 — Questionnaire: Term low birth weight study, Wolaita Sodo. (DOCX 23 kb) [file 12884_2018_1789_MOESM1_ESM.docx]

**Questionnaire:**

This questionnaire is prepared to gather information on mothers and their newborns socio-demographic, obstetric, maternal morbidity, health facility utilization, dietary habit and iron folate supplementation and anthropometric characteristics

| Note the inclusion criteria   - Is the child born single? 1. Yes 2. No - Is the child free of any visible major birth defect 1. Yes 2. No - Is the birth live birth? 1. Yes 2. No - Is the gestational age of the pregnancy known? 1. Yes 2. No   If “No” to any of the inclusion criteria, stop the data collection.  Circle on the answer option selected | | | | | | | | | | | | | | | |
| --- | --- | --- | --- | --- | --- | --- | --- | --- | --- | --- | --- | --- | --- | --- | --- |
| Health facility type/code | | | | | |  | | | | | | | | | |
| **Section I. Newborn’s characteristics** | | | | | | | | | | | | | | | |
| **S.N** | | **Questions** | | | | **Answers** | | | | | | **Skip to** | | | |
| 101 | | Birth weight of the baby in grams | | | | ____________ grams | | | | | |  | | | |
| 102 | | What is the sex of the newborn? | | | | 1. Male 2. Female | | | | | |  | | | |
| **Section II. Sociodemographic characteristics of the mother** | | | | | | | | | | | | | | | |
| 201 | | How old are you? | | | | | Age in completed years ______ | | | | | |  | | |
| 202 | | What is your current marital status? | | | | | 1. Single 2. Married 3. Divorced/separated 4. Widowed | | | | | |  | | |
| 203 | | What is your educational status? | | | | | 1. No formal education 2. Primary education (1-8) 3. Secondary education (9-12) 4. College and above | | | | | |  | | |
| 204 | | What is your occupation? | | | | | 1. Housewife 2. Government employee 3. Private employee 4. Merchant 5. Other (specify)__________ | | | | | |  | | |
| 205 | | Where is your residence? | | | | | 1. Rural 2. Urban | | | | | |  | | |
| 206 | | How much is the monthly income of the household in Ethiopian birr? | | | | | ___________________ | | | | | |  | | |
| **Section III. maternal obstetric characteristics** | | | | | | | | | | | | | | | |
| 301 | How many times did you give birth? | | | | | | | | 1. First time 2. Twice 3. Three times 4. Four times 5. Five and above | | | |  | | |
| 302 | What is the time interval between the current baby and his/her immediate elder? | | | | | | | | 1. Primipara 2. _______ months | | | |  | | |
| 303 | Have you ever had a pregnancy that ended up in miscarriage before 7 months? | | | | | | | | 1. Yes 2. No | | | |  | | |
| 304 | Have you ever had a pregnancy that ended up in stillbirth? | | | | | | | |  | | | |  | | |
| 305 | Have you had a small baby in previous births? | | | | | | | | 1. Yes 2. No | | | |  | | |
| **Section IV. Maternal morbidity characteristics** | | | | | | | | | | | | | | | |
| 401 | Do you have previous history of hypertension? | | | | | | | | 1. Yes 2. No | | | |  | | |
| 402 | Do you have previous history of diabetes mellitus? | | | | | | | | 1. Yes 2. No | | | |  | | |
| 403 | Have you had abnormal medical conditions during this pregnancy? | | | | | | | | 1. Yes 2. No | | | |  | | |
| **Section V. Health facility related characteristics** | | | | | | | | | | | | | | | |
| 501 | Did you visit any health facility for ANC service for the current pregnancy? | | | | | | | | 1. Yes 2. No | | | |  | | |
| 502 | How many times did you receive ANC during your current pregnancy? | | | | | | | | ________ times | | | |  | | |
| 503 | How many months pregnant were you when you first received ANC service for your current pregnancy? | | | | | | | | _________ months | | | |  | | |
| 504 | During (any of) your current ANC visit (s), did you get dietary counseling? | | | | | | | | 1. Yes 2. No | | | |  | | |
| **Section VI. Dietary habit and iron folate supplementation** | | | | | | | | | | | | | | | |
| 601 | During the current pregnancy, did you take any additional meal than usual? | | | | | | | | 1. Yes 2. No | | | |  | | |
| 602 | During the current pregnancy, were you given or did you buy iron tablets? | | | | | | | | 1. Yes 2. No | | | | If no, skip to Q 701 | | |
| 603 | Did you take/consume the tablets? | | | | | | | | 1. Yes 2. No | | | | If no, skip to Q 701 | | |
| 604 | How many iron tablets did you take? | | | | | | | | __________ tablets | | | |  | | |
| **Section VII. Dietary intake characteristics** | | | | | | | | | | | | | | | |
| **Food type** | | | **Daily** | **4-6*/week** | | | | **1-3*/week** | | **1-3*/month** | **Once/month** | | | | **Never** |
| **Cereals** | | | | | | | | | | | | | | | |
| Barely flour roasted | | |  |  | | | |  | |  |  | | | |  |
| Porridge | | |  |  | | | |  | |  |  | | | |  |
| Maize boiled | | |  |  | | | |  | |  |  | | | |  |
| Maize roasted | | |  |  | | | |  | |  |  | | | |  |
| Maize unleavened bread | | |  |  | | | |  | |  |  | | | |  |
| Enjera | | |  |  | | | |  | |  |  | | | |  |
| Bread | | |  |  | | | |  | |  |  | | | |  |
| **Roots and tubers** | | | | | | | | | | | | | | | |
| False banana root baked | | |  |  | | | |  | |  |  | | | |  |
| Refined f. banana porridge | | |  |  | | | |  | |  |  | | | |  |
| Sweet potato boiled | | |  |  | | | |  | |  |  | | | |  |
| Potato sauce | | |  |  | | | |  | |  |  | | | |  |
| Yam boiled | | |  |  | | | |  | |  |  | | | |  |
| Maize+false banana baked | | |  |  | | | |  | |  |  | | | |  |
| **Legumes** | | | | | | | | | | | | | | | |
| Chick pea roasted | | |  |  | | | |  | |  |  | | | |  |
| Chick pea sauce | | |  |  | | | |  | |  |  | | | |  |
| Pea flour sauce | | |  |  | | | |  | |  |  | | | |  |
| Lentil sauce | | |  |  | | | |  | |  |  | | | |  |
| **Dairy products** | | | | | | | | | | | | | | | |
| Fresh cow’s milk | | |  |  | | | |  | |  |  | | | |  |
| Butter cow’s milk | | |  |  | | | |  | |  |  | | | |  |
| Sour cow’s milk | | |  |  | | | |  | |  |  | | | |  |
| Cottage cow’s cheese | | |  |  | | | |  | |  |  | | | |  |
| **Meat** | | | | | | | | | | | | | | | |
| Raw beef minced | | |  |  | | | |  | |  |  | | | |  |
| Beef liver raw | | |  |  | | | |  | |  |  | | | |  |
| Beef raw | | |  |  | | | |  | |  |  | | | |  |
| Beef grilled | | |  |  | | | |  | |  |  | | | |  |
| Tripe beef raw | | |  |  | | | |  | |  |  | | | |  |
| Chicken roasted | | |  |  | | | |  | |  |  | | | |  |
| **Fish** | | | | | | | | | | | | | | | |
| Fish boiled | | |  |  | | | |  | |  |  | | | |  |
| **Vegetables** | | | | | | | | | | | | | | | |
| Kale boiled | | |  |  | | | |  | |  |  | | | |  |
| Cabbage boiled | | |  |  | | | |  | |  |  | | | |  |
| Swiss chard boiled | | |  |  | | | |  | |  |  | | | |  |
| Pumpkin boiled | | |  |  | | | |  | |  |  | | | |  |
| Tomato boiled | | |  |  | | | |  | |  |  | | | |  |
| **Fruits** | | | | | | | | | | | | | | | |
| Avocado | | |  |  | | | |  | |  |  | | | |  |
| Banana | | |  |  | | | |  | |  |  | | | |  |
| Guava | | |  |  | | | |  | |  |  | | | |  |
| Lemon | | |  |  | | | |  | |  |  | | | |  |
| Mango | | |  |  | | | |  | |  |  | | | |  |
| Orange | | |  |  | | | |  | |  |  | | | |  |
| Papaya | | |  |  | | | |  | |  |  | | | |  |
| Pineapple | | |  |  | | | |  | |  |  | | | |  |
| **Nuts** | | | | | | | | | | | | | | | |
| Peanut roasted | | |  |  | | | |  | |  |  | | | |  |
| **Egg** | | | | | | | | | | | | | | | |
| Egg whole boiled | | |  |  | | | |  | |  |  | | | |  |
| Egg whole fried with fat | | |  |  | | | |  | |  |  | | | |  |
| **Home-made non-alcoholic beverages** | | | | | | | | | | | | | | | |
| Coffee | | |  |  | | | |  | |  |  | | | |  |
| Emmer wheat gruel | | |  |  | | | |  | |  |  | | | |  |
| Tea | | |  |  | | | |  | |  |  | | | |  |
| **Home-made alcoholic beverages** | | | | | | | | | | | | | | | |
| Mead | | |  |  | | | |  | |  |  | | | |  |
| Tella | | |  |  | | | |  | |  |  | | | |  |
| Shameta | | |  |  | | | |  | |  |  | | | |  |
| Caticala | | |  |  | | | |  | |  |  | | | |  |
| **Commercial alcoholic beverages** | | | | | | | | | | | | | | | |
| Alcoholic beverages | | |  |  | | | |  | |  |  | | | |  |
| **Commercial soft drinks** | | | | | | | | | | | | | | | |
| Soft drinks | | |  |  | | | |  | |  |  | | | |  |
| **Section VIII. Anthropometric characteristics of the study participants** | | | | | | | | | | | | | | | |
| **S.N** | | **Question** | | | **Answer** | | | | | | | | | **Remark** | |
| 801 | | MUAC of the mother | | | __________ centimeters | | | | | | | | |  | |
